# Single-Mode Ring Resonator-Based Optomechanical Transducers for Advanced Atomic Force Sensing

**DOI:** 10.1021/acsphotonics.5c01914

**Published:** 2025-11-10

**Authors:** Yide Zhang, Artem S. Vorobev, Savda Sam, S. Hadi Badri, Mauro David, Bernhard Lendl, Georg Ramer, Liam O'Faolain

**Affiliations:** † Institute of Chemical Technologies and Analytics, 27259 TU Wien, Vienna 1060, Austria; ‡ Centre for Advanced Photonics and Process Analysis, 587895 Munster Technological University, Cork T12P928, Ireland; § Tyndall National Institute, Cork T12R5CP, Ireland; ∥ Institute of Solid State Electronics, TU Wien, Vienna 1040, Austria; ⊥ Christian Doppler Laboratory for Advanced Mid-Infrared Laser Spectroscopy in (Bio-)process Analytics, TU Wien, Vienna 1060, Austria

**Keywords:** optomechanical transducer, ring resonator, silicon photonics, Atomic Force Microscopy (AFM), displacement densing, force sensing

## Abstract

Atomic force microscopy (AFM) is a widely used technique for high-resolution imaging and force sensing, yet its performance is fundamentally constrained by the cantilever size, spring constants, and mechanical frequencies. To overcome these limitations, we present a compact and highly efficient single-mode ring resonator-based optomechanical transducer on an silicon-on-insulator (SOI) platform. Unlike conventional designs that rely on whispering gallery modes (WGMs) resonators, our approach ensures mode stability, facilitates straightforward signal interpretation, and enhances measurement reliability by eliminating mode-splitting effects and complex optical responses. Coupled with a picogram-scale cantilever, our system achieves exceptional displacement resolution of 6.7 × 10^–16^ m/Hz^1/2^ and force detection down to 5.0 × 10^–14^ N, providing a high-performance alternative to existing optomechanical AFM transducers. The tunable mechanical resonance frequency (1.3 to 22.5 MHz) and adjustable stiffness (0.46 to 3.54 N/m) enable precise force sensing across a broad range of applications, from soft matter characterization to high-speed imaging. Importantly, our results exhibit strong agreement with theoretical predictions, ensuring accurate and direct displacement measurements. Our results establish this single-mode optomechanical transducer as a robust, high-sensitivity platform for next-generation AFM and nanoscale sensing applications, offering a compact, scalable, and highly precise alternative to traditional free-space optical detection methods. The combination of high displacement resolution, mode stability, and tunable performance establishes this optomechanical transducer as a promising advancement in integrated nanoscale sensing and AFM applications.

## Graphical abstract

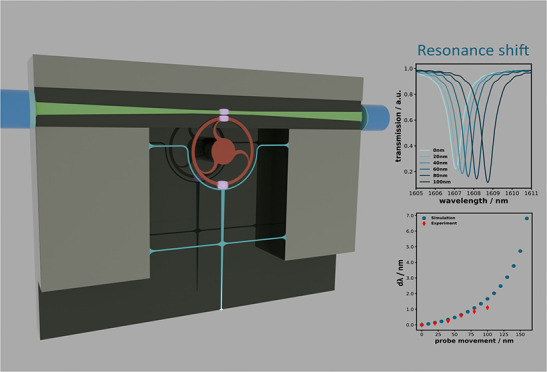

## Introduction

Atomic Force Microscopy (AFM)^1,2^ has become an essential tool for high-resolution imaging and force sensing across diverse fields, including nanotechnology, materials science, and biology. Unlike optical and electron microscopy, AFM relies on physical interactions^3–8^ between a nanoscale probe and a sample surface, enabling nanoscale characterization of mechanical,^9^ electrical,^10^ biological,^11^ and thermal^12^ properties. Furthermore, when combined with pulsed IR lasers for sample excitation, AFM can also achieve chemical characterization^13,14^ with nanoscale resolution. However, AFM performance is fundamentally constrained by the mechanical properties of the cantilever, particularly its resonance frequency, stiffness, and displacement detection method.

Traditional optical-lever detection methods, which track cantilever deflection via laser reflection onto a position-sensitive photodiode,^2^ remain widely used due to its sensitivity. However, these approaches rely on bulky free-space optics and expensive tunable lasers, while remaining prone to alignment challenges and environmental disturbances.^15,16^ The time resolution of AFM is fundamentally constrained by the thermal limit of the cantilever,^17^ necessitating low mass and high quality factors to minimize thermal noise off resonance.^18^ To achieve high resonant frequencies (>1 MHz), which are essential for faster imaging and rapid force spectroscopy,^19,20^ cantilever miniaturization is often required. However, the resulting reduced dimensions are limited by optical diffraction,^21,22^ further making displacement detection and signal stability challenging.

To address these limitations, integrated cavity optomechanics^23–25^ have emerged as a powerful alternative. By leveraging the interaction between a high-quality optical resonator and a mechanical oscillator, this approach enables displacement transduction with sensitivities close to the quantum limit.^26–28^ In optomechanical systems, evanescent wave coupling allows precise motion detection,^29^ making them well-suited for AFM applications.

Despite their advantages, prior optomechanical AFM transducers face several key challenges. Microdisk-based transducers^30,31^ achieve high optical Q-factors but rely on multimode whispering gallery modes (WGMs), which introduce mode splitting, unpredictable optical responses, and complex signal interpretation.

A ring-shaped resonator with a mechanical probe directly attached to the optical ring, supporting WGMs was employed in a prior optomechanical AFM design.^32^ Designs in this category have enabled high-frequency optomechanical sensors with mechanical frequencies exceeding 100 MHz and exceptional displacement sensitivity.^33^ Their fixed ultrahigh stiffness (40 kN/m) can be a limitation for probing soft materials in conventional AFM. Although high-stiffness probes have achieved excellent resolution under high-frequency, high-Q operation^34^ and in noncontact AFM modes,^35^ such stiffness is less optimal for standard contact-mode measurements on compliant samples.

While WGM-based devices have shown successful applications as optomechanical AFM probes, they typically support multiple optical modes and often experience power-dependent thermal shifts, mode crowding, and high sensitivity to temperature variations.^36–38^

In this work, we present a single-mode ring resonator-based optomechanical transducer on a silicon-on-insulator (SOI) platform. Our design ensures spectral isolation and reduced mode competition, enabling more stable and reproducible displacement sensing. It supports both direct displacement measurement with fast and high-accuracy sensing (200 nm dynamic range) and an AFM feedback-assisted mode for structured samples requiring larger displacement tracking.^39,40^ Our device achieves a displacement resolution below femtometer/Hz^1/2^ (often termed displacement sensitivity in the AFM/optomechanics literature^30,33^ ) and force detection in the tens of femtonewtons range, while maintaining tunable mechanical frequencies from 1.3 to 22.5 MHz and adjustable stiffness from 0.46 to 3.54 N/m by modifying the ring resonator geometry.

This new design shows promise as a compact and versatile alternative to free-space optical detection of cantilever displacement, making it adaptable to a wide range of sensing applications. By demonstrating a strong correlation between experimental optical resonance shifts and theoretical predictions, this work establishes the SOI-based optomechanical transducer as a viable solution for high-frequency probe applications, thereby advancing optomechanical AFM sensing and expanding its applicability in material characterization, photothermal imaging, and biological research.

## Results and Discussion

### Device Design and Simulation

The device is fabricated on an SOI wafer, consisting of a 220 nm top silicon layer, 3 μm silicon dioxide underlayer, and a 725 μm silicon handle layer. Fabrication was carried out using electron beam lithography (EBL), with process details provided in Methods section.

A scanning electron microscope (SEM) image of a fabricated device is shown in Figure 1a. The structure consists of a tapered waveguide that evanescently couples light into and out of a single-mode optical microring resonator. The narrowest section of the tapered waveguide, as well as the ring width, is 500 nm, ensuring support for a single TE mode. The device operates at telecom wavelengths and under critical coupling conditions with a 60 nm gap between the ring and tapered waveguide (gap_rw_). This gap was initially determined through finite-difference time-domain (FDTD) simulations during the design process and later validated experimentally. To optimize light coupling efficiency, gap_rw_ was finely adjusted in 10 nm steps, ranging from 50 to 100 nm, as detailed in Supporting Information Section S1.

**Figure 1:**
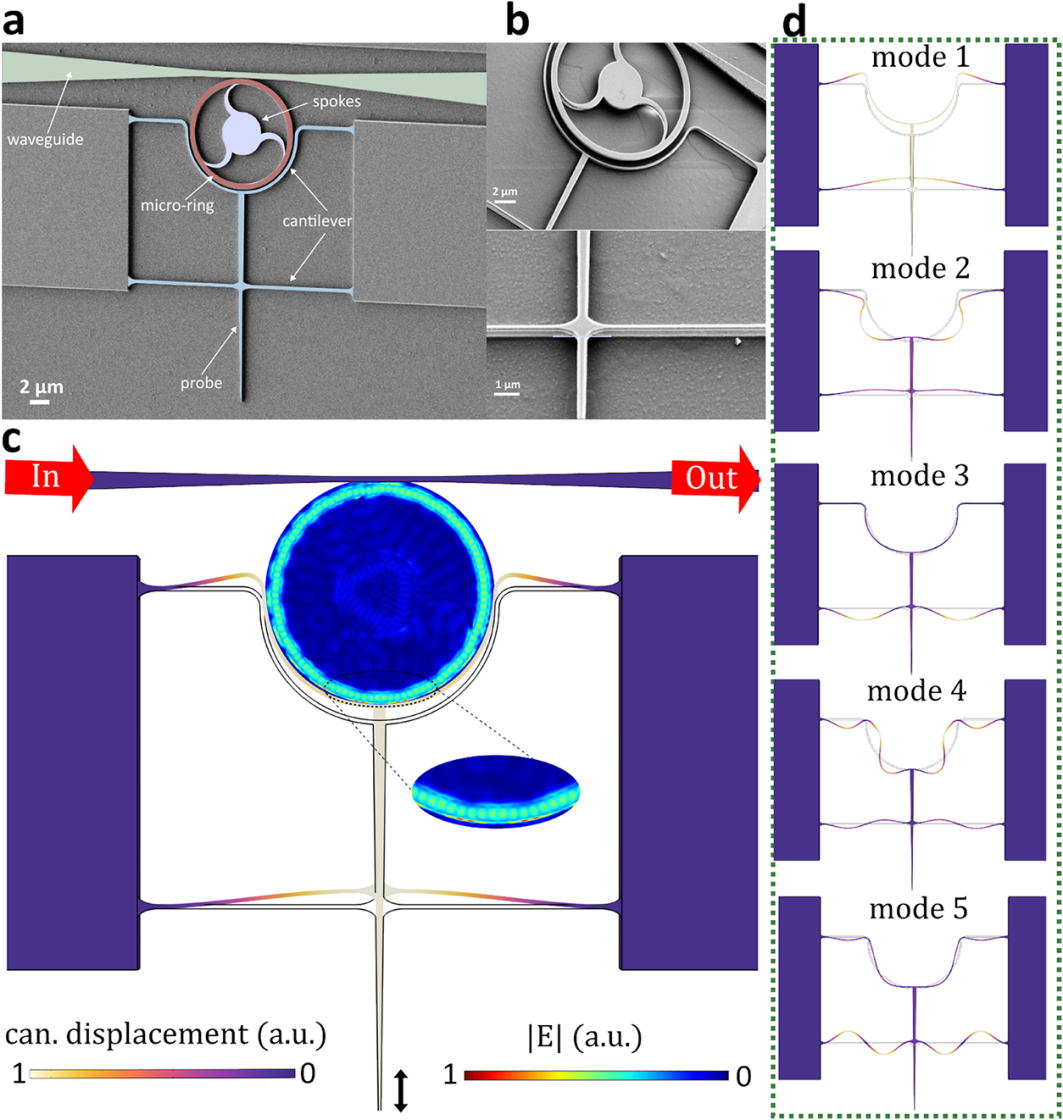
(a) False-colored SEM image of the microring cantilever with a 5 μm ring radius, characterized in this study to illustrate the device geometry. The applied color scheme distinctly highlights the waveguide, microring, supporting spokes, and cantilever, enhancing structural clarity. (b) Magnified SEM micrograph showing the suspended cantilever in greater detail. (c) Illustration of the devices working principle. The gray regions represent the device in its equilibrium state, while the colored regions depict the deformed shape of the microring cantilever in its first in-plane mechanical mode, as simulated via FEM. The microring has a 5 μm radius and a 500 nm width, supported by three curved spokes. The cantilever is connected via two 5 μm arms, with a probe width of 100 nm and a length of 20 μm. The entire device has a thickness of 220 nm. A color map within the ring resonator cavity shows the normalized electric field amplitude (|*E*|) at a resonance wavelength of 1555.14 nm, calculated using FDTD simulations, with the cantilever positioned 50 nm away. The cantilever’s motion (can. displacement) modulates the optical mode of the resonator, enabling signal transduction. (d) Simulated first five in-plane mechanical modes of the cantilever, with amplitudes scaled by a factor of 10,000 for visual clarity.

The blue rendering in Figure 1a illustrates the flexible cantilever-probe unit, which is positioned adjacent to the ring resonator in the fabricated optomechanical transducer. The cantilever part consists of both semicircular and straight sections. The semicircular section, curved around the edge of the ring, features a width and a gap between the ring and the cantilever (gap_rc_) of 200 nm to maximize interaction with the optical ring. gap_rc_ determines the dynamic range of the cantilever’s movement relative to the ring, enabling direct measurement compared to previous designs^30,33,41^ that rely on AFM feedback. This design choice allows for faster signal tracking and a simpler readout.

The cantilever is clamped at both ends and includes a 20 μm-long probe, supported by a straight cantilever positioned 10 μm away from the probe tip. The probe width is chosen to be 100 nm for mechanical characterization; however, it can be thinned down to tens of nanometers for high spatial resolution microscopy.^42^ This design ensures the cantilever remains free-standing, allowing optimized in-plane motion for strong coupling with the ring resonator.^30^

The cantilever is released through selective etching of the sacrificial oxide layer down to a thickness of 700 nm using buffered HF (7:1 ratio) (see Figure S4). The undercutting process is carefully controlled to ensure free vibration of the cantilever, while keeping the microring and tapered waveguide securely anchored to the substrate. The suspended cantilever is shown in Figure 1b. To improve mechanical stability, the microring is supported by three curved spokes, which have been optimized to minimize optical scattering losses.

The cantilever deforms under applied force at the tip or vibrates due to thermal-mechanical noise. In both cases, the gap between the cantilever and the ring changes (gap_rc_ changes), causing a local variation in the effective refractive index near the ring, as demonstrated in Figure S5. This variation directly affects the propagation of optical modes at the rings edge, leading to an optical resonance shift as well as a corresponding intensity change. Figure S7 presents an FDTD simulation illustrating the optical modes of the device without the cantilever. When the cantilever is positioned 50 nm from the ring resonator, its presence modifies the optical modes, as depicted in Figure 1c. Notably, Figure 1c is plotted at a fixed wavelength of 1555.14 nm, while a supplementary video^43^ spanning the spectral range from 1540 to 1560 nm further elucidates the optical mode evolution, revealing the response of both the ring resonator and the inner spokes.

The mechanical modes of the cantilever are analyzed using finite-element method (FEM) simulations, with the first five modes shown in Figure 1d. Since our design primarily utilizes in-plane motion, we focus on even-symmetry in-plane modes, which exhibit strong coupling with the optical modes of the ring resonator. These modes are particularly relevant to AFM applications, enabling high-sensitivity force and displacement sensing.

### Optomechanical Detection

The fabricated devices are characterized using a home-built optomechanical setup, schematically shown in Figure 2. This setup allows for the characterization of multiple devices on the same chip, while simultaneously collecting optical spectra and mechanical resonance data. A tunable laser (Santec TSL-570), operating over a wide wavelength range of 1480 to 1640 nm, is used for excitation. The laser light is polarization-controlled before being coupled into a microlensed fiber, which injects the light into the device. After passing through the device, the light is collected and split into two branches.

**Figure 2:**
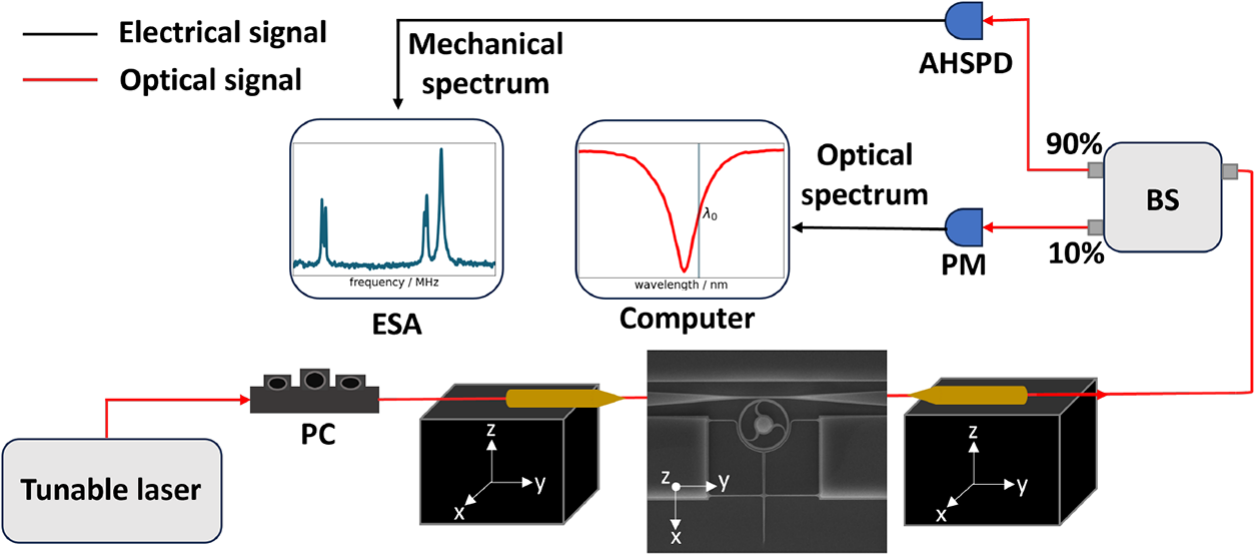
Schematic of the characterization setup: A power-controlled near-infrared tunable laser first passes through a polarization controller (PC) before being coupled into and collected from the devices using microlensed fibers. These fibers are mounted on three-dimensional nanopositioning stages, integrated with a custom-built visualization system. The inset displays an optical image captured using this system. After collection, the light is split in a 90:10 ratio using a fiber beam splitter (BS): 10% of the light is directed to a multichannel power meter (PM) to a computer for optical power monitoring. The remaining 90% is sent to an amplified high-speed photodetector (AHSPD) and then to an electrical spectrum analyzer (ESA) to extract the devices mechanical response.

10% of the collected light is directed to a multiport optical power monitor (Santec MPM-215), which is interfaced with computer-controlled software to record optical spectra. After identifying an optical mode with a high optical quality factor (*Q*_o_), a wavelength is selected at the inflection point of the individual cavity resonance (highlighted in Figure 3a,c,e) – corresponding to the steepest slope of the transmission dip – to maximize sensitivity to mechanical vibrations. The remaining 90% of the transmitted signals is sent to an amplified high-speed photodetector (FPD510-FC-NIR, Thorlabs Inc.). This signal is then analyzed using an electrical spectrum analyzer (FSV4, Rohde & Schwarz GmbH), providing direct access to the thermo-mechanical resonance spectrum of the cantilever.

**Figure 3:**
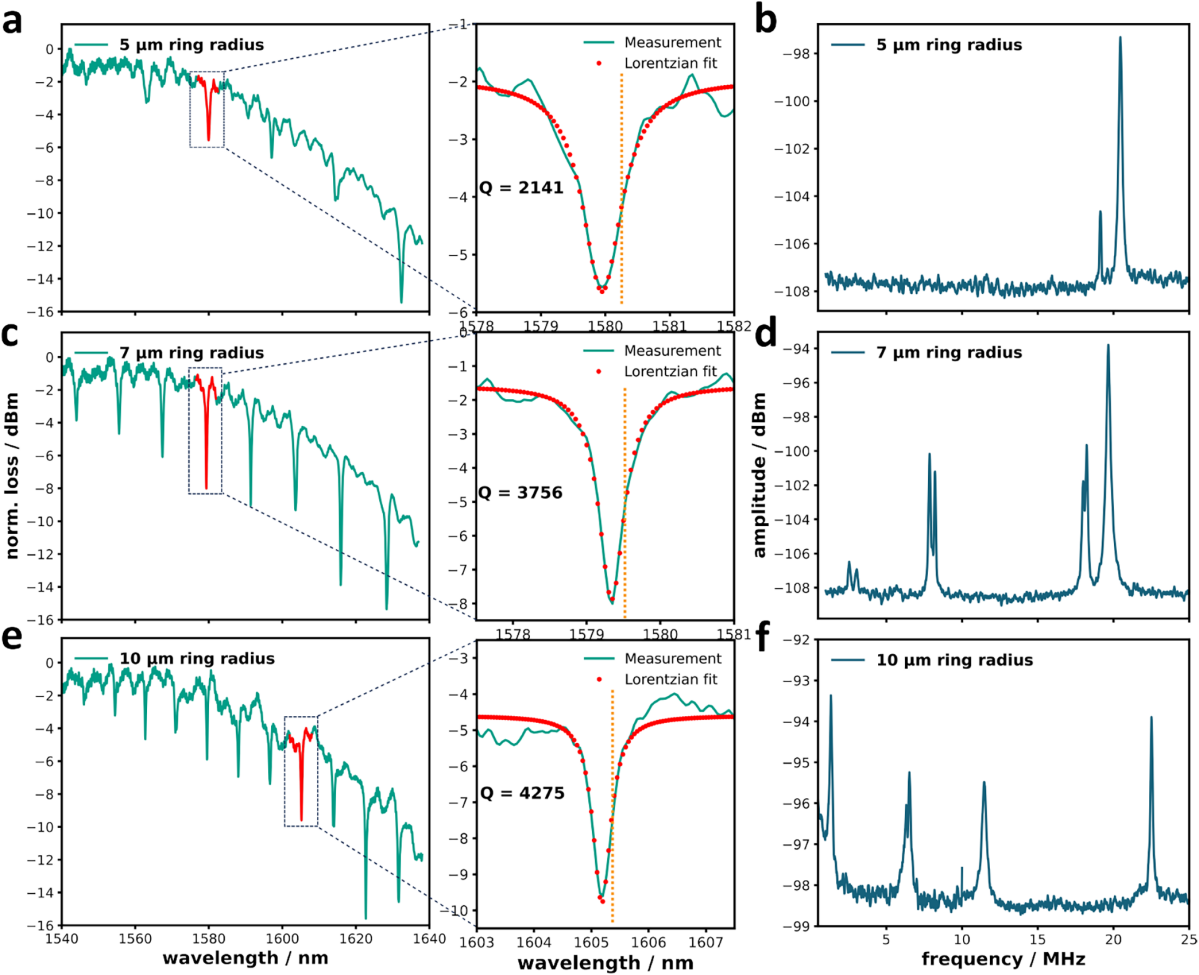
Optical and mechanical spectra of devices with different ring radii: Panels (a), (c), and (e) display the optical spectra for devices with ring radii of 5, 7, and 10 μm, respectively. Panels (b), (d), and (f) show the corresponding mechanical spectra for the same devices. The right-hand plots in (a), (c), and (e) provide zoomed-in views of the red traces from the left-hand plots. Orange dashed lines in the zoomed-in figures indicate the wavelengths used for mechanical spectrum measurements.

Using the optomechanical setup described above, we performed air-based measurements on devices with ring radii of 5, 7, and 10 μm. In these experiments, thermal noise induces random oscillations of the cantilever, which occur at its mechanical resonance frequencies. The normalized transmission spectra across the full wavelength range for these devices are shown in Figure 3a,c,e, along with zoomed-in scans of individual optical resonances.

The *Q*_o_ was extracted by fitting the experimental spectra with Lorentzian functions. The measured *Q*_o_ values are 2141, 3756, and 4275 for the 5 μm, 7 μm, and 10 μm rings, respectively, showing an increase in *Q*_o_ with ring size. This trend is attributed to reduced bending losses in larger rings, whereas smaller rings experience higher bending losses.^44^

To determine the response of the transducer, the laser was tuned to the inflection point of the TE cavity mode, as shown in the zoomed-in spectra of Figure 3a,c,e. The corresponding mechanical spectra covering 1 to 25 MHz are presented in Figure 3b,d,f. The observed peaks arise from thermally driven cantilever motion, as confirmed by FEM simulations. These peaks primarily correspond to the first five in-plane even-symmetry modes, as illustrated in Figure 1d. In the 5 μm device, the first two mechanical modes are not clearly visible. This is attributed to their higher mechanical stiffness (as shown in Figure S6), which leads to reduced thermal displacement amplitudes, as well as weaker modulation of the optical gap.

Moreover, FEM simulations indicate that the mechanical frequency of the device decreases as the size of the ring increases. The measured frequencies from the examined devices, shown in Figure 4, align well with the theoretical predictions. Some predicted modes are not observed experimentally due to their low thermomechanical amplitudes and weak optical coupling, which result in signals below the detection threshold of our setup. Mode 2, in particular, is weaker than Modes 1 and 3 because its mode profile introduces a node near the optical coupling region (as shown in Figure 1d). In the 7 μm device, the overlap is still sufficient to produce a detectable but relatively weak signal, whereas in the 10 μm device the geometric shift of the mode shape reduces the overlap further, leaving Mode 2 below the detection threshold.

**Figure 4:**
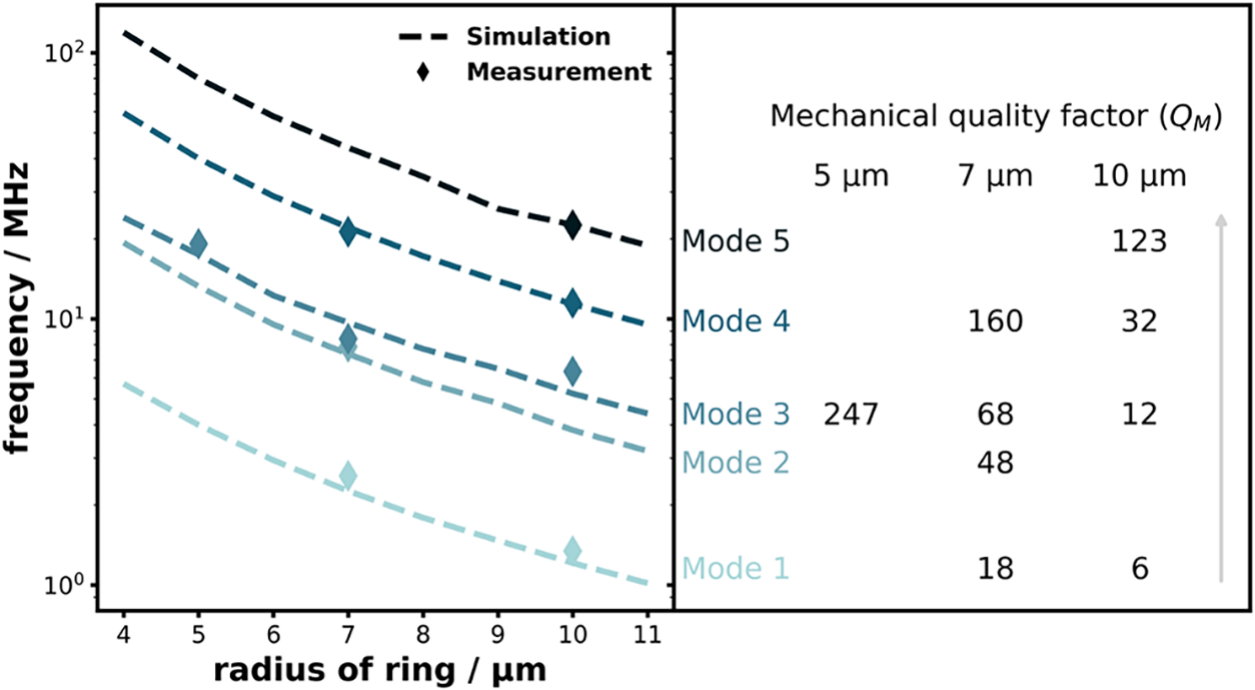
Left panel: Simulated mechanical frequencies for devices with ring radii ranging from 4 to 11 μm, alongside experimentally measured results from the tested devices. Right panel: Calculated mechanical quality factors (*Q*_M_) corresponding to each vibrational mode.

The measured mechanical quality factors (*Q*_M_) values for these modes range from approximately *Q*_M_ ≈ 6 for mode 1 of the 10 μm device to *Q*_M_ ≈ 247 for mode 3 of the 5 μm device. Additionally, for each individual device, we observe that *Q*_M_ increases for higher-order modes.

Notably, mechanical simulations were performed on the full device geometry, including the ring, spokes, and cantilever. Although the complete structure supports additional mechanical modes, our measured resonance spectra are notably clean, showing only a few dominant peaks that match well with the simulated cantilever in-plane modes. This confirms that the optical signal modulation is primarily driven by the cantilever’s in-plane vibrations, which modulate the lateral gap between the cantilever and the ring waveguide. The contribution of ring and spoke modes is negligible under our operating conditions

### Transduction Efficiency

To further evaluate the capability of our devices as displacement and force sensors, we modified the optomechanical setup by incorporating a three-dimensional closed-loop controlled piezo stage (MAX381/M, Thorlabs Inc.) with a 3D-printed probe holder mounted on top. A straight tungsten probe with a 500 nm tip diameter (Micro Support Co., Ltd.) was inserted into the probe holder and positioned at a 15° angle relative to the device surface, as shown in Figure 5a. This angle was chosen to ensure that the tip contacted only the cantilever without touching the substrate or surrounding structures. The probe position was precisely controlled using a stepper motor, allowing us to approach the probe tip to the end of the cantilever.

**Figure 5:**
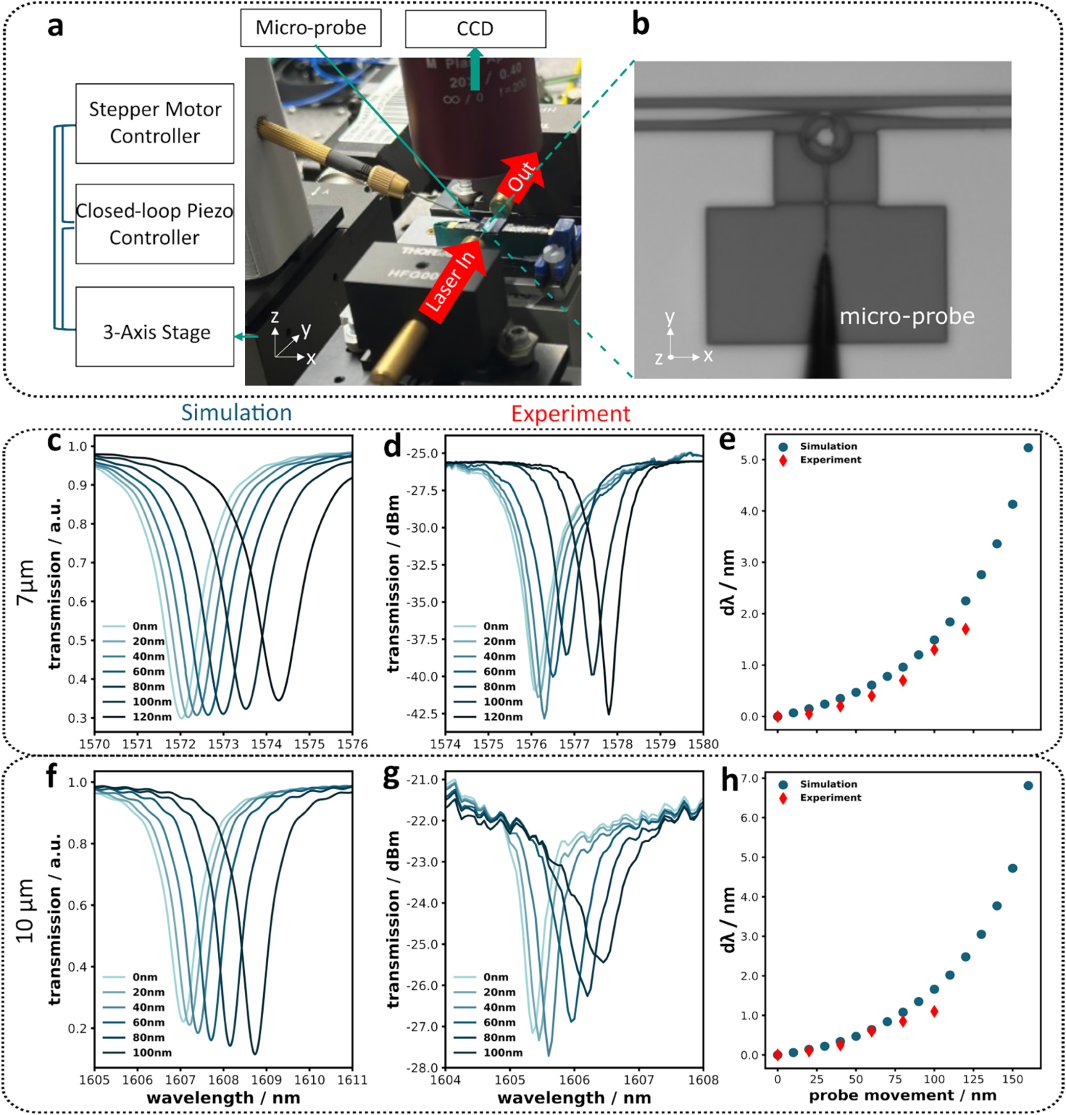
(a) Microprobe measurement setup used for the mechanical characterization of microring cantilevers. (b) Optical image captured using the custom-built visualization system, showing a fine probe in contact with the microring cantilever. (c, f) FDTD-simulated optical spectra depicting the effect of cantilever motion toward the ring resonator in 20 nm steps, for devices with ring radii of 7 and 10 μm, respectively. (d, g) Corresponding experimental results validating the simulated optical response. (e, h) Resonance wavelength shift (Δ*λ*), obtained from Lorentzian fitting of each transmission spectrum and referenced to the equilibrium state (0 nm cantilever motion), plotted as a function of probe displacement.

Transmission spectra were recorded before probe contact, labeled as “0 nm” in Figure 5d,g. After making contact, the probe incrementally displaced the cantilever in 20 nm steps with transmission spectra collected at each step (Figure 5d, 7 μm device). As the probe pushed the cantilever closer to the ring, the optical resonance shifted toward longer wavelengths. The observed resonance shift, Δ*λ*, was obtained by Lorentzian fitting of each transmission spectrum, with the fitted center wavelength referenced to the equilibrium state and is observed to increase with probe displacement. This resonance shift is caused by evanescent coupling between the ring resonator and the cantilever (Figure S3). As the cantilever approaches, the overlap with the ring’s evanescent field modifies the effective refractive index. The experimental results align closely with simulations, confirming the capability to use the developed device. Notably, the cantilever is displaced by an external probe, which introduces locally nonuniform deformation which can produce nonmonotonic changes in dip depth and line width. Our calibration uses Lorentzian fits to the resonance center, so these variations do not affect the extracted wavelength shift that defines the dynamic range.

Based on the results shown in Figures 5e,h, the optomechanical coupling factor was determined through FEM simulations, confirmed by experimental findings. This factor is defined as

1 $$g_{\mathrm{OM}}/2\pi =\frac{d\omega_{O}}{\mathrm{dx}}$$

where *d*ω_O_ is the angular optical cavity mode frequency shift and *dx* represents the cantilever displacement. For the probe displacement range in this study, *g*_OM_/2π varies from 0.3 to 1.7 GHz/nm for the 7 μm device, and from 0.6 to 1.3 GHz/nm for the 10 μm device. The experimentally achieved values are consistent with previously reported *g*_OM_/2π values for silicon microdisk AFM probes,^30^ which were based solely on simulations. Furthermore, *g*_OM_/2π increases as the gap between the ring and cantilever decreases, confirming the strong optomechanical interaction in our system.

The minimum detectable force *F*_min_ is calculated based on^45^

2 $$F_{\min}=\sqrt{\frac{4kk_{B}\mathrm{TB}}{2\pi f_{M}Q_{M}}}$$

where *B* is the measurement bandwidth, *T* the temperature (300 K), and cantilever stiffness *k* and *k*_B_ is the Boltzmann constant.

The stiffness of the optomechanical cantilever depends on the ring size, with values of 0.46 N/m for 5 μm device and 3.54 N/m for 10 μm, as shown in Figure S6. Taking a common bandwidth of *B* = 50 Hz, consistent with prior studies for comparison to other experiments,^30,45^ the calculated *F*_min_ for the first mode of the 7 μm device is 5.0 × 10^–14^ N. For the 10 μm device, *F*_min_ is 2.4 × 10^–13^ N. This 5-fold increase is consistent with the stiffness-dependent force detection limit, where higher stiffness reduces mechanical responsiveness to weak forces.

The device’s displacement measurement sensitivity is calibrated using thermal-mechanical noise measurements. The detection resolution limit is 6.7 × 10^–16^ m/Hz^1/2^ and 8.3 × 10^–16^ m/Hz^1/2^ for the 7 and 10 μm devices, respectively, with detailed calculations provided in the Methods section.

The achieved displacement and force resolution closely align with previously reported values^30,33^ (see comparison table in Supporting Information Section S2), demonstrating comparable or improved resolution while maintaining a large dynamic measurement range. Furthermore, unlike previous studies that relied on controlled nitrogen or vacuum environments, our device achieves its sensitivities in ambient air and improves upon other high-sensitivity optomechanical AFM probes used for AFM-IR^41^ by more than an order of magnitude, underscoring its robustness for real-world AFM applications. Such fast, highly sensitive probes are essential for detecting weak interactions in nanoscale systems-from transient thermal phenomena and thermal conductivity imaging to van der Waals forces and single-molecule interactions.

## Conclusion and Discussion

In summary, we have developed and demonstrated a novel optomechanical device based on an SOI platform, integrating optical microring resonators with suspended cantilevers. The device operates under a single TE mode, ensuring stable optical performance with high wavelength control precision. Through a combination of FDTD and FEM simulations, supported by experimental validation, we characterized the optical and mechanical properties of devices with varying ring sizes, confirming their suitability for high-precision sensing applications.

The device exhibits strong optomechanical coupling, achieving a balance between moderate optical quality factors. Experimentally, the optomechanical coupling factor *g*_OM_/2π ranges from 0.3 to 1.7 GHz/nm, aligning well with simulated values. This strong coupling enables the optical modes to transduce the cantilever’s megahertz-frequency motion with a displacement resolution of 6.7 × 10^–16^ m/Hz^1/2^ while operating in ambient air. The measured optical quality factors *Q*_o_ are primarily limited by scattering from sidewall roughness arising from nonoptimized fabrication. While very high *Q*_o_ can in theory improve displacement resolution, they also narrow the resonance line width and increase sensitivity to environmental fluctuations. The present values therefore represent a practical balance between resolution, dynamic range, and operational robustness. The mechanical quality factors *Q*_M_ and stiffness values were characterized, revealing clear dependencies on device size and mode order. The measured *Q*_M_ values are modest, likely limited by air damping and anchor losses.^46^ Although high *Q*_M_ improves thermal-noise-limited resolution, it also increases ring-down time and requires larger spacing between excitation pulses. The moderate *Q*_M_ values observed here thus represent a practical trade-off, providing sufficient force resolution while enabling faster response for time-resolved AFM applications. Furthermore, the minimum detectable force was calculated to be as low as 5.0 × 10^–14^ N (in 50 Hz bandwidth), underscoring the device’s capability for high-precision displacement and force sensing.

These findings establish our optomechanical transducer as robust platforms for high-precision sensing, leveraging the synergy between integrated photonics and nanomechanics. The demonstrated performance paves the way for advancements in high-throughput scanning and integrated photonic-based metrology.

While this work demonstrates optical readout of mechanical motion via direct mechanical actuation, quantitative detection and calibration of noncontact forces remain future goals. The device supports noncontact actuation methods such as electrostatic or tip–sample forces, and forthcoming studies will focus on implementing calibrated force sensing to obtain first force measurements. Future work will focus on full AFM integration, which requires cantilever release and extension beyond the chip edge so that the probe can overhang for sample access. Such processes have been demonstrated in recent work,^47^ but we also envision exploring alternative fabrication strategies to establish a robust and scalable release process. Together with fiber bonding for optical access and AFM-IR driven excitation at mechanical resonances, these developments will enable advanced AFM imaging applications

## Methods

### Detection Sensitivity

The thermal-mechanical spectra are shown in Figure 3b,d,f. The cantilever is driven by the thermal noise of room temperature. The displacement sensitivity induced by the thermal noise can be described by^31,48^

3 $${\left[S_{\mathrm{xx}}\left(\omega \right)\right]}^{1/2}={\left(\frac{4k_{B}T\omega_{M}/Q_{M}}{m_{\mathrm{eff}}\left({\left(\omega^{2}-\omega_{M}^{2}\right)}^{2}+\omega^{2}\omega_{M}^{2}/Q_{M}^{2}\right)}\right)}^{1/2}$$

where ω_M_ is angular mode frequency (2π*f*_M_). The effective mass for the fundamental mode is *m*_eff_ = 0.73*m*_0_.^49^ *m*_0_ = 11.45 pg for the 7 μm device and *m*_0_ = 14.26 pg for the 10 μm device. These values have been observed from FEM simulations. The limit of detection resolution is 6.7 × 10^–16^ m/Hz^1/2^ and 8.3 × 10^–16^ m/Hz^1/2^ for 7 and 10 μm device, respectively. The thermal-mechanical displacement is [*S*_*xx*_(ω)­Δ*f*]^1/2^,^49^ where Δ*f* is the measurement bandwidth.

### Simulation

The optical ring resonator and cantilevers were designed using the Python Gdspy module, and the resulting designs were exported as .gds files. These files were utilized for optical transmission simulations, solid mechanical simulations, and final fabrication, ensuring consistency across all stages of the process.

Optical transmission simulations were conducted using finite-difference time-domain (FDTD) methods with a 3D-FDTD commercial software package from Ansys, Inc. To optimize simulation performance, a quasi-2D simulation approach was employed within the 3D-FDTD solver. This approach enhances computational efficiency while preserving simulation accuracy. Mechanical mode and stiffness simulations of the cantilevers were conducted using the finite element method (FEM) with the 3D Solid Mechanics module in COMSOL Multiphysics software 6.2.

The FDTD simulations for the cantilever transduction efficiency were conducted under static conditions by varying the gap between the cantilever and the ring.

### Device Fabrication

A thermally oxidized bulk Silicon wafer with a deposited layer of poly-Si (220 nm thick) from the SOITEC Corp. was utilized. Initially, a ZEP 520A resist layer (450 nm thick) was spin-coated onto the wafer at 190 °C for 3 min. The desired device layouts were then defined on the resist using electron beam lithography (EBL) with a voltage of 100 kV and 1 nA current, followed by development in a bath of *n*-Amyl Acetate solution for 90 s and rinsing with IPA. Subsequently, the patterns were transferred to the SOI layer through an inductively coupled plasma (ICP) etching process using O_2_:CHF_3_ chemistry in 8:42 ratio (with an etch rate of approximately 35 nm/min). The remaining resist mask was removed via an O_2_ plasma ashing step followed by immersion in MICROPOSIT 1165 remover for 10 min. Finally, a cleaning cycle involving Piranha etching, Acetone, and IPA were conducted to conclude the fabrication process step.

For the tip releasing, an additional EBL exposure step was implemented. PMMA-A14 resist layer (2 μm thick) was spin-coated onto the sample and soft-baked at 150 °C for 3 min. The desired opening regions were then defined on the resist using second stage alignment EBL (50 nm alignment error) with a voltage of 100 kV and 10 nA current, followed by development in a bath of MIBK and IPA solution for 120 s and rinsing with IPA with following by a 150 °C 20 min hard baking step. The SiO_2_ layer in the opening regions was partially etched using the buffered hydrofluoric acid solution with a 7:1 ratio. The etching process was conducted for 7 min at an approximate etch rate of 100 nm/min. Finally, a cleaning cycle was performed, consisting of a 10 min immersion in acetone to dissolve the PMMA protection layer, followed by rinsing with IPA for thorough cleaning.

## Supplementary Material



## Notes

Section S1: Gap distance optimization (additional figures) and Section S2: Comparison table (Table S1). Code and raw data to generate all figures is available on Zenodo (DOI:10.5281/zenodo.15077551) (PDF)
