# Supplementary material for: Single-Mode Ring Resonator-Based Optomechanical Transducers for Advanced Atomic Force Sensing
Source: ACS Photonics. 2025 Nov 10;12(12):6778–87. doi: 10.1021/acsphotonics.5c01914 (PMC12715834; doi:10.1021/acsphotonics.5c01914)
Supplement: Supplementary file 1 [file ph5c01914_si_001.pdf]

SUPPLEMENTARY INFORMATION

Single-Mode Ring Resonator-Based Optomechanical Transducers for Advanced Atomic Force Sensing

Yide Zhang, Artem S.Vorobev, Savda Sam, S. Hadi Badri, Mauro David, Bernhard Lendl, Georg Ramer* and Liam O'Faolain*

E-mail: georg.ramer@tuwien.ac.at;William.Whelan-Curtin@mtu.ie

Y.Zhang, S.Sam, B.Lendl, G.Ramer

Institute of Chemical Technologies and Analytics, TU Wien, Getreidemarkt 9 /E164-02-1, Vienna, 1060,Austria

Y.Zhang, A.S.Vorbev, S.Sam, S.H.Badri, L.O'Faolain

Centre for Advanced Photonics and Process Analysis, Munster Technological University,Rossa Avenue, Bishopstown, Cork, T12P928, Ireland

M.David

Institute of Solid State Electronics, TU Wien, Gusshausstrasse 25-25a / E362, Vienna, 1040, Austria

G.Ramer

Christian Doppler Laboratory for Advanced Mid-Infrared Laser Spectroscopy in (Bio-)process Analytics, TU Wien, Vienna, Austria

Table of Contents

S1 Gap distance optimization **S3**

S2 Comparison table **S11**

References **S11**

S1 Gap distance optimization

To optimize the gap distance between the tapered waveguide and micro-ring (gap_{rw}), we fabricated devices with gaps in 10 nm steps, ranging from 50 nm to 100 nm. The optical transmission measurements were conducted on the optomechanical setup with 100% power (10 mW) directed to the chip and collected afterwards by multichannel power meter.

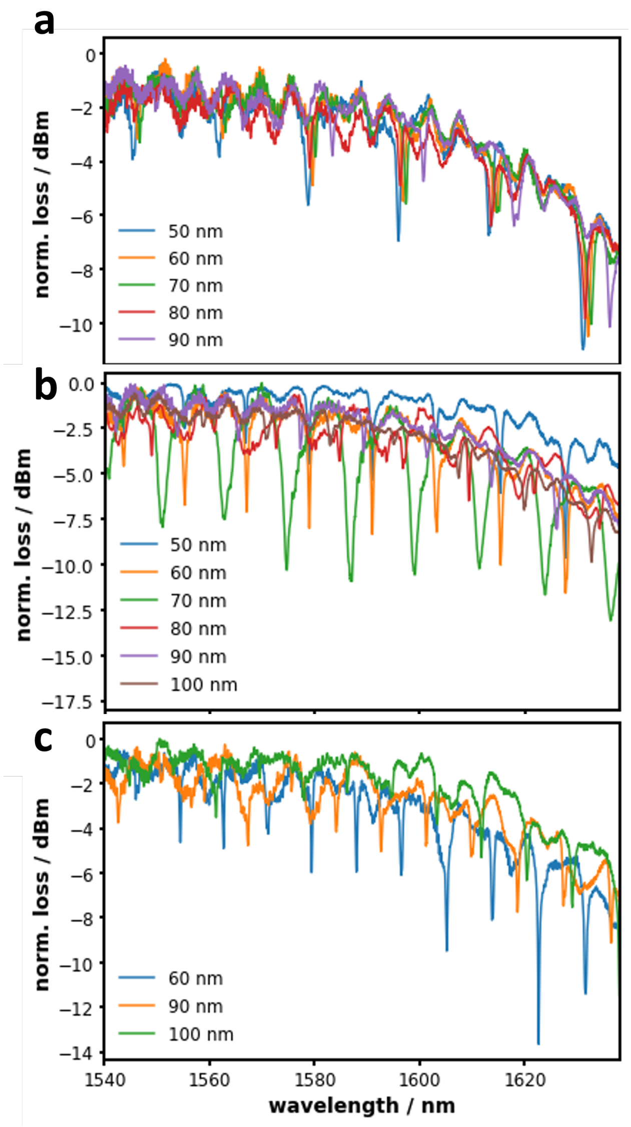

Figure S1: Gap optimization measurements for examined gaps ranging from 50 nm to 100 nm on: (a) 5 µm device, (b) 7 µm device, and (c) 10 µm device.

The resonance position is primarily determined by the effective optical path length of the cavity, which depends on the ring radius and the effective refractive index of the guided mode. While the ring radius remains constant in our design, the evanescent field profile and the mode overlap with the tapered waveguide vary with the coupling gap. These variations cause slight perturbations to the guided mode, altering the effective refractive index and resulting in small shifts in the resonance wavelength.

To clarify this point, we have added additional simulations in the Supporting Information. These examine the effect of varying the coupling gap between the tapered waveguide on a 10 μm-radius ring from 60 nm to 100 nm in 10 nm. As shown in Figure S2, the resonance position exhibits a clear blue shift with increasing gap, consistent with the expected reduction in modal overlap and effective index.

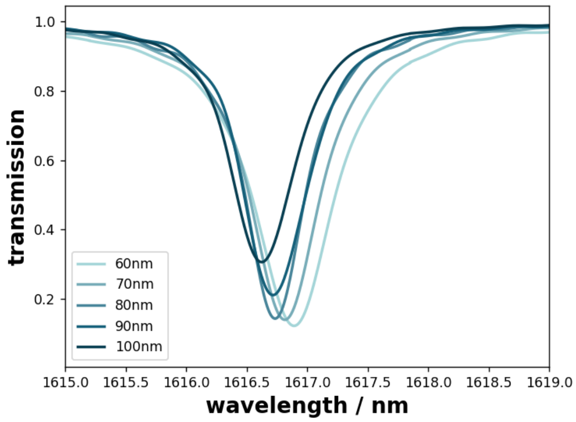

Figure S2: Simulated transmission spectra of a 10 μm-radius ring resonator with the cantilever positioned 200 nm away, showing the effect of varying the coupling gap between the ring and the tapered waveguide. The coupling gap is varied from 60 nm to 100 nm in 10 nm steps. A systematic blue shift in the resonance wavelength is observed with increasing gap, attributed to reduced evanescent field overlap and a corresponding decrease in the effective refractive index.

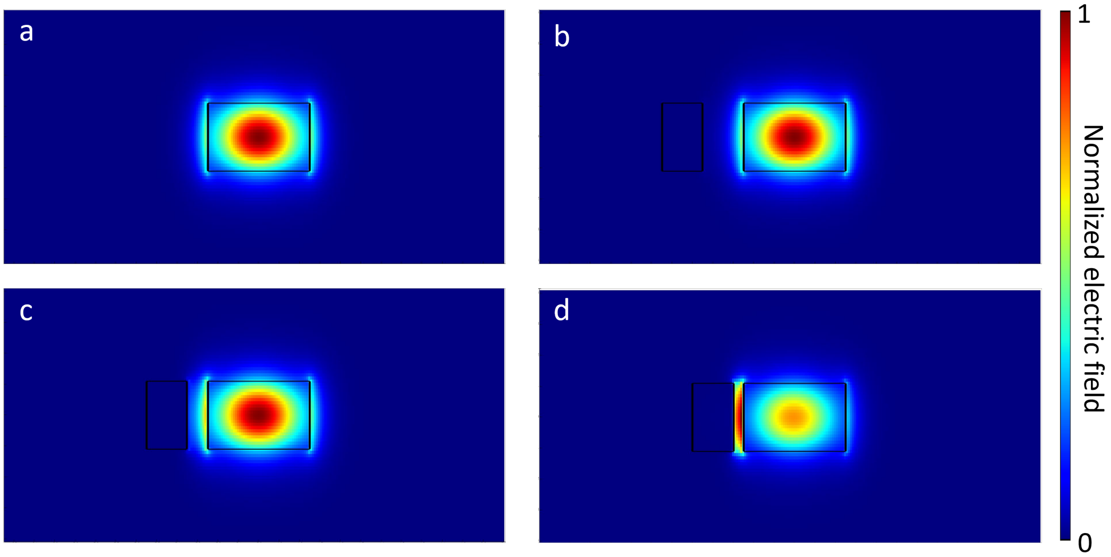

Figure S3: Simulated electric field intensity profiles (z–y cross-section) of the ring resonator and the semi-circular section of the cantilever. (a) Ring waveguide only (no cantilever). (b) Cantilever positioned 200 nm from the ring. (c) Cantilever positioned 100 nm from the ring. (d) Cantilever positioned 50 nm from the ring.

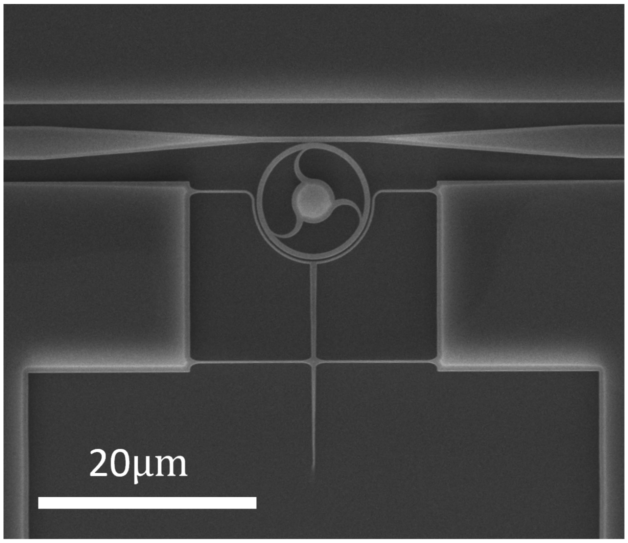

Figure S4: An SEM image of the micro-ring cantilever, with a ring radius of 5 μm, taken after HF etching

We define the dynamic range of the device (200 nm) as the maximum variation in the cantilever–ring gap over which the optical resonance can be tracked unambiguously without active feedback control. This parameter is determined by the interplay between coupling efficiency and resonance shift: if the gap becomes too large, the perturbation of the optical mode is too weak to produce a measurable resonance change. Figure S4c shows the simulated effective refractive index as a function of the cantilever–ring gap. The results indicate that the optical mode remains measurably perturbed for gap variations up to approximately 200 nm, beyond which the coupling becomes too weak for reliable tracking. The theoretical prediction is consistent with the experimental data in the main text (Figs. 5e,h), where resonance wavelength shifts are observed over the same displacement range

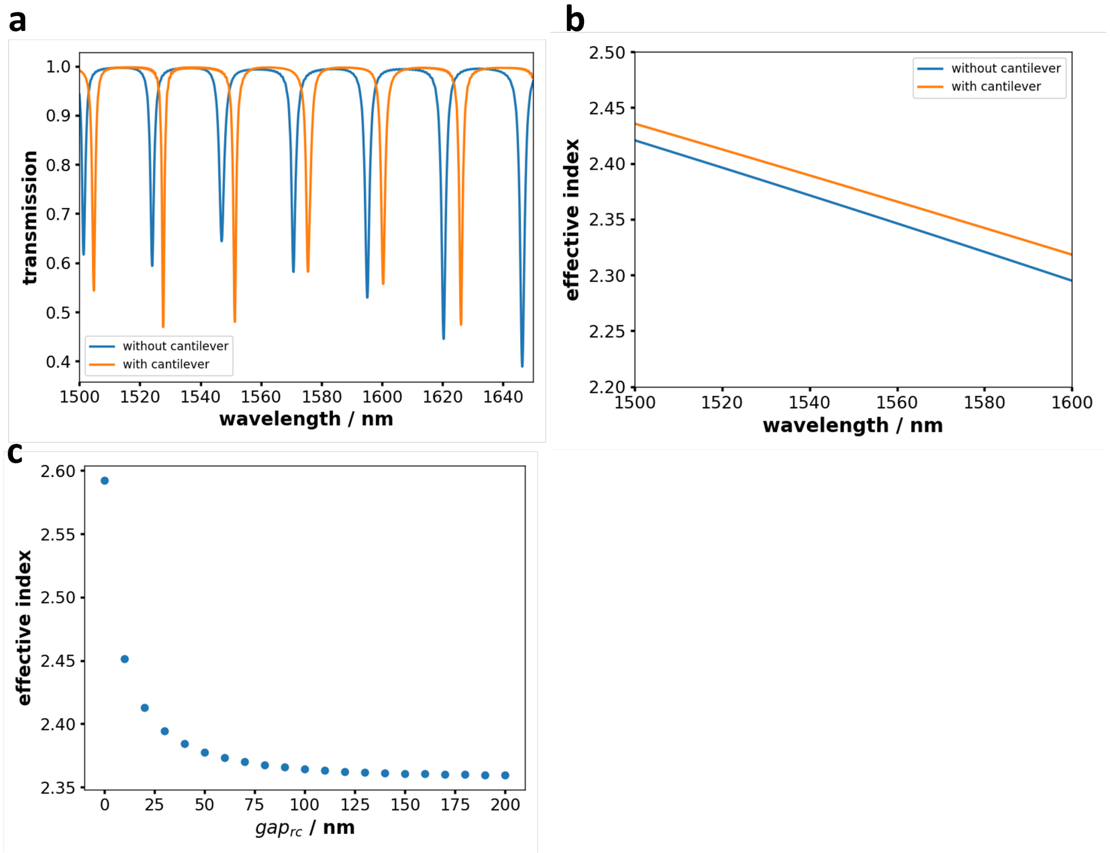

Figure S5: Optical response and mode sensitivity analysis of the 5 µm-radius ring device. (a) Transmission spectra of the 5 µm-radius ring resonator with and without ($gap_{rc} = 50\,\text{nm}$) the cantilever. The comparison highlights the cantilever's influence on the optical resonance, showing a red-shift in the spectral response when the cantilever is present. (b) Effective index as a function of wavelength (1500–1600 nm) for two configurations: (i) without the cantilever and (ii) with the cantilever positioned at a 50 nm gap from the ring. This plot illustrates the modal dispersion and demonstrates how the cantilever affects the guided mode. (c) Effective index as a function of the gap between the cantilever and the ring waveguide. A decreasing gap leads to stronger mode-cantilever interaction, resulting in a pronounced increase in effective index.

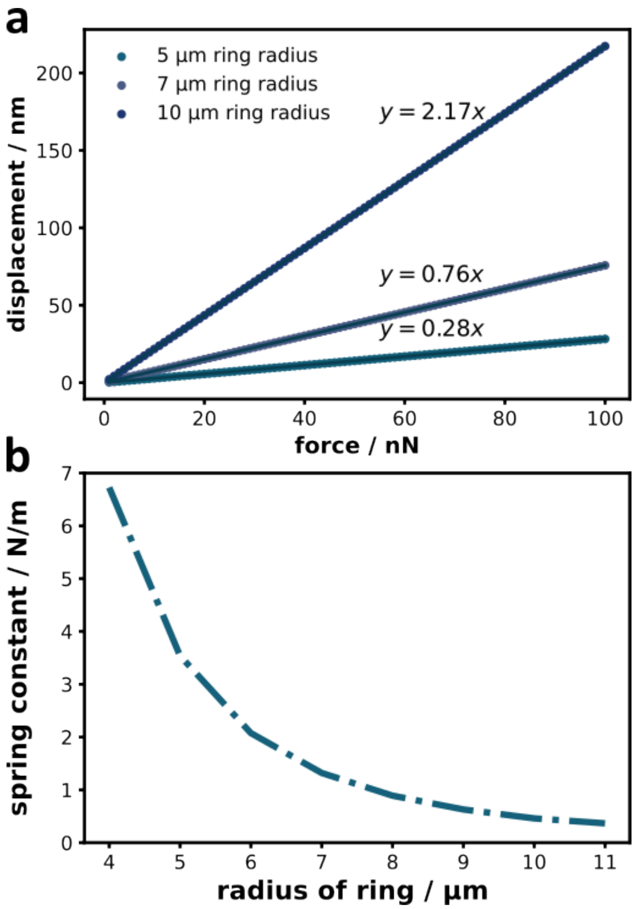

Figure S6: (a) Simulation of the cantilever's in-plane displacement under different applied forces for the three examined ring sizes: 5 µm, 7 µm, and 10 µm. Solid lines represent the linear fitted curves, with the corresponding fitted equations displayed above the solid lines. (b) Calculated spring constants of the cantilever based on simulation and linear fitting of the displacement/force curve. The spring constants are equivalent to the reciprocal of the slope of the linear displacement/force curve.

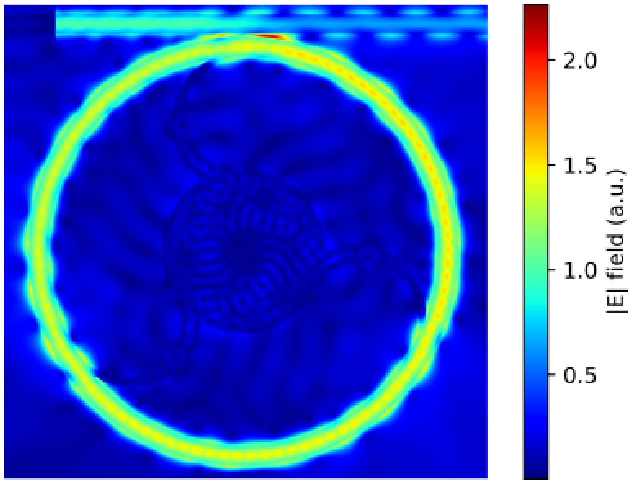

Figure S7: Electric field amplitude of a 5 µm ring with supporting spokes at resonance, calculated using a 2.5D varFDTD simulation.

S2 Comparison table

Table S1: Comparison of the performance of our single-mode ring resonator-based optomechanical transducer with other whispering gallery mode (WGM)-based optomechanical AFM transducers.

**References**	**Displacement Sensitivity** ($m/Hz^{1/2}$)	**Force Sensitivity** (N)	**Stiffness** (N/m)
**Srinivasan et al.**[S1](in N_2)	4.4×10^{-16}	5.1×10^{-14}	$0.1 - 10$
**Chae et al.**[S2] (in air)	3.0×10^{-15}	Not stated	Not stated
**Schwab et al.**[S3] (in air)	3.0×10^{-16}	Not stated	40,000
**This work**(in air)	6.7×10^{-16}	5.0×10^{-14}	$0.46 - 3.54$

References

[S1] Srinivasan, K.; Miao, H.; Rakher, M. T.; Davanço, M.; Aksyuk, V. Optomechanical Transduction of an Integrated Silicon Cantilever Probe Using a Microdisk Resonator. *Nano Letters* **2011**, *11*, 791–797.

[S2] Chae, J.; An, S.; Ramer, G.; Stavila, V.; Holland, G.; Yoon, Y.; Talin, A. A.; Allendorf, M.; Aksyuk, V. A.; Centrone, A. Nanophotonic Atomic Force Microscope Transducers Enable Chemical Composition and Thermal Conductivity Measurements at the Nanoscale. *Nano Letters* **2017**, *17*, 5587–5594.

[S3] Schwab, L.; Allain, P. E.; Mauran, N.; Dollat, X.; Mazenq, L.; Lagrange, D.; Gély, M.; Hentz, S.; Jourdan, G.; Favero, I.; Legrand, B. Very-high-frequency probes for atomic force microscopy with silicon optomechanics. *Microsystems & Nanoengineering* **2022**, *8*, 32.
